# Apoptotic pathways in pancreatic ductal adenocarcinoma

**DOI:** 10.1186/1476-4598-7-64

**Published:** 2008-07-24

**Authors:** Rainer Hamacher, Roland M Schmid, Dieter Saur, Günter Schneider

**Affiliations:** 1 II. Medizinische Klinik, Klinikum rechts der Isar, Technische Universität München, München, Germany

## Abstract

Pancreatic ductal adenocarcinoma (PDAC) is one of the most common causes of cancer related death. Despite the advances in understanding of the molecular pathogenesis, pancreatic cancer remains a major unsolved health problem. Overall, the 5-year survival rate is less than 5% demonstrating the insufficiency of current therapies.

Most cytotoxic therapies induce apoptosis and PDAC cells have evolved a plethora of molecular mechanisms to assure survival. We will present anti-apoptotic strategies working at the level of the death receptors, the mitochondria or involving the caspase inhibitors of the IAP family. Furthermore, the survival function of the phosphotidylinositol-3' kinase (PI3K)/AKT- and NF-kappaB-pathways are illustrated. A detailed molecular knowledge of the anti-apoptotic mechanisms of PDAC cells will help to improve therapies for this dismal disease and therapeutic strategies targeting the programmed cell death machinery are in early preclinical and clinical development.

## Introduction

Pancreatic ductal adenocarcinoma (PDAC) is one of the most malignant tumor with an unfavorable prognosis. More than 30.000 people develop pancreatic adenocarcinoma each year in the United States, and almost all are expected to die from the disease. Although the incidence of pancreatic cancer is only about 10 in 10^5^, it is the fourth male and female leading cause of cancer-related death [1]. One reason for the poor prognosis of PDAC is the insensitivity to most therapies like chemotherapy, radiotherapy and immunotherapy. Therefore, surgical resection offers at the moment the only potential chance for cure. The 5-year survival rate of all patients is below 5%, and the median survival time after diagnosis is 6 months. Furthermore, only about 20% of patients curative resected survive longer than 5 years [2].

The hallmarks of nearly all cancers are deregulation of the cell cycle machinery, self-sufficiency in growth signals, insensitivity to growth inhibitory signals, evasion of apoptosis, tissue invasion, metastasis and sustained angiogenesis [2-4]. These characteristic changes can also be found in PDAC and are detailed discussed in excellent reviews from the groups of Depinho, Maitra, Hruban and Friess [5-7]. The demonstration that the fraction of apoptotic cells in PDAC predicts overall survival shows the important contribution of the apoptotic machinery towards the tumorbiology of PDAC [8].

Most chemotherapies act by induction of apoptosis. Therefore, evasion of apoptosis is mainly responsible for the insufficiency of current therapies [9]. Tumor cells use multiple pathways to escape apoptosis [10]. This review will focus on the deregulation of apoptotic pathways in PDAC.

### Apoptosis – An overview

Apoptosis or programmed cell death is a central regulator of normal tissue homeostasis. The physiological "cell suicide" program is essential for the elimination of redundant, damaged and infected cells [11,12]. Disturbed apoptosis is involved in the pathogenesis of multiple diseases, especially cancer.

Execution of apoptosis relies on a group of cysteine proteases, the caspases [13]. Caspases are synthesized as pro-forms and become activated by cleavage next to aspartate residues. Since caspases cleave and activate each other, an amplification mechanism through a protease cascade exists, assuring proper execution of apoptotic cell death [13]. In addition, caspases cleave numerous substrates, like nuclear lamins, inhibitors of DNase or cytoskeletal proteins, ultimatively leading to the typical morphological alterations of apoptosis [13].

**Figure 1:**
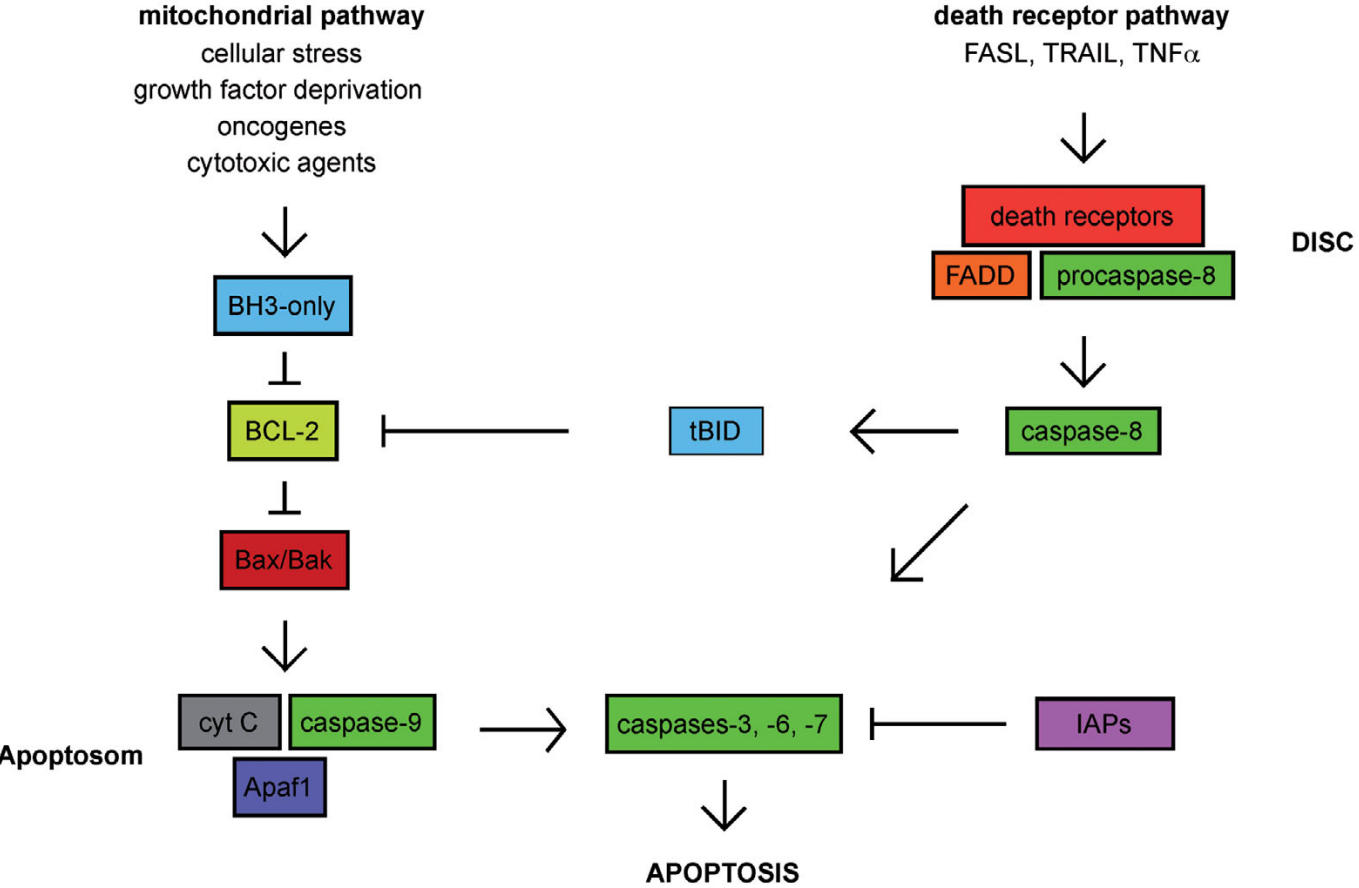
**Pathways to Apoptosis**. The mitochondrial pathway is activated by BH3-only proteins, which sense cellular stress and inactivate pro-survival BCL-2 family members. This leads to the permeabilization of the outer mitochondrial membrane and the release of cyctochrom C, Apaf-1 and caspase-9. The death receptor pathway is activated by the TNF family ligands. Caspase-8 is activated by adaptor proteins including FADD. In PDAC cells, the death receptor pathway is linked to the mitochondria by the BH3-only protein Bid that is cleaved by caspase-8.

There are two alternative pathways to initiate apoptosis and both finally activate the executioner caspases-3, -6 and -7 (figure 1):

The first pathway is called intrinsic or mitochondrial pathway, because the mitochondria takes the key position by initiating apoptosis. The exact mechanism of initiation by different apoptotic stimuli is still not entirely clear, but involves an imbalance of pro- and anti-apoptotic members of the BCL-2 protein family [14]. This imbalance finally leads to the activation of the pro-apoptotic BCL-2 family members BAX and/or BAK and the perturbance of the integrity of the outer mitochondrial membrane [14]. This induces the release of cytochrom c and other apoptotic regulators, like apoptosis-inducing factor (AIF), Smac (second mitochondria-derived activator of apoptosis)/DIABOLO (direct inhibitor of apoptosis protein (IAP)-binding protein with low PI), endonuclease G or Omi/HtrA2 from the intermembraneous space of mitochondria [15]. In the cytosol, cytochrom c, APAF-1, ATP and the initiator procaspase-9 are forming the apoptosome multiprotein complex and activate the initiator caspase-9. This induces the cleavage of the executioner caspases, like caspase-3. Furthermore, the potent endogenous inhibitors of caspases, the inhibitor of apoptosis proteins (IAPs) are neutralized by Smac/DIABOLO or Omi/HtrA2 [16].

The second pathway is called the extrinsic pathway and is mediated by different death receptors on the cell surface [17-19]. These receptors are members of the tumor-necrosis factor (TNF) receptor superfamily, including the TNF-, FAS-(APO-1, CD95) and TRAIL-(TNF-related apoptosis inducing ligand) receptors. They share a common intracellular domain, which is called death domain. Activation of the receptors after extracellular binding of the specific ligands (TNF-α, FAS-L and TRAIL) initiates the recruitment of FADD (FAS-associated death domain protein), procaspase-8 and -10 to the death domain, which are forming the DISC (death inducing signaling complex) [20]. At the DISC, the initiator caspase-8 is activated. In type I cells the activated initiator caspases are sufficient to induce executioner caspases directly. In contrast type II cells need the signal enhancing-effect of mitochondria to induce apoptosis. Here, caspase-8 cleaves the pro-apoptotic BH3-only BCL-2 family member BID, which translocates to the mitochondrial membrane and induces the release of apoptogenic factors from the mitochondria [11]. Pancreatic carcinoma cells are type II cells [21,22]. The type I/II concept, is a good example of the crosstalk between extrinsic and intrinsic apoptotic signaling and demonstrates the complexity of apoptosis [23].

### Apoptosis and Carcinogenesis of PDAC – an under-investigated field

Apoptosis is a fundamental anti-neoplastic mechanism in normal cells to prevent tumorigenesis. Nearly all neoplastic changes during the development of a normal cell to a cancer cell, like DNA-damage, oncogene activation or cell cycle deregulation, are potent inducers of the programmed cell death pathway. Therefore, overcoming the apoptotic failsafe is observed in many cancers as discussed by Lowe, Cepero and Evan [24].

In accordance with the multi-step theory of carcinogenesis, the natural history of PDAC seems to gradually evolve through precursor lesions, the so-called **pan**creatic **i**ntraepithelial **n**eoplasias (PanIN) 1 to 3 to invasiv PDAC [2,5-7]. Whereas the progressive accumulation of genetic somatic mutations, including mutations in the oncogene *K-RAS *and the tumor suppressors *CDKN2A*, *TP53*, *SMAD4/DPC4 *and *BRCA2 *are quite good understood, the contribution of apoptosis to carcinogenesis of PDAC is not well defined [3]. In PanIN 1 and 2 lesions, no apoptotic cells could be detected, arguing for the contribution of anti-apoptotic strategies early in the carcinogenesis of PDAC [25]. These anti-apoptotic strategies most likely enable cell survival under oncogenic stress conditions [25]. Recently, two members of the IAP (inhibitors of apoptosis) protein family, cIAP2 and Survivin were shown to be early overexpressed during carcinogenesis of PDAC. One anti-apoptotic function of the IAP family is dependent on the inhibition of caspase activity [16]. Early overexpression of cIAP2 was demonstrated in 30% of low-grade PanIN lesions, 50% of high grade PanIN lesions and 85% of PDAC [26]. In addition to cIAP2, Survivin was shown to be overexpressed already in PanIN1 lesions at the mRNA and protein level [27]. Although the observed overexpression of cIAP2 and Survivin, suggests early contribution in tumorigenesis of PDAC, molecular pathways engaged by these molecules in preneoplastic lesions of the pancreas are unknown.

### Death receptor signaling – blocked and abused

Many PDAC cells are characterized by a high IC_50 _for death receptor mediated apoptosis, although they express the corresponding receptors and the execution machinery of apoptosis is intact [22,28-32]. PDAC cells have evolved resistance mechanisms, which are especially working at the death receptor level resulting in DISC insufficiency [33].

The protein tyrosine phosphatase FAP-1 (FAS-associated phosphatase-1) is overexpressed in PDAC and protects pancreatic carcinoma cells from FAS-mediated apoptosis by inhibiting the activation of caspase-8 [28,34]. It seems that FAP-1 interferes with the translocation of CD95 to the cell surface. This leads to a low receptor density on the cell surface and to a disruption of receptor trimerization, which is necessary to form the DISC. Furthermore a direct inhibition of caspase-8 by FAP-1 is discussed [34]. The resistance towards FAS-mediated apoptosis enables PDAC cells to express simultaneously FASL, which might contribute to escape the immunosurveillance. Compatibly, expression of FASL was observed in 6/6 investigated PDAC cell lines and co-culture of PDAC cells with T-cells results in T-cell death in a FasL-dependent fashion [28].

Another potent inhibitor of caspase-8 activation is c-FLIP_L_, which is a structural homologue of caspase-8, lacking amino acid residues that are critical for caspase activity. c-FLIP_L _competes with procaspase-8 for binding to FADD at the DISC [35,36]. Together with FAP-1, c-Flip_L _is highly expressed in PDACs [35]. In addition to high c-FLIP_L _expression, low amounts of FADD were shown to contribute to resistance against CD95L- and TRAIL-induced apoptosis in PDAC cells [32].

A further important mechanism to escape death-receptor induced apoptosis is to favor pro-survival signaling after triggering the death receptor. In PDAC cells, especially the activation of NF-κB signaling (see below) contributes to the resistance towards death receptor induced apoptosis [21]. One factor involved in the induction of pro-survival signaling is the death receptor adaptor TRAF2. TRAF2 belongs to the TRAF-family of adaptor proteins which are involved in the signal transduction pathways of the TNF-receptor family. Kalthoff's group observed TRAF2 overexpression in 94% of PDAC and the FASL-resistant cell lines PancTuI, Panc89 and Panc1 [37]. Furthermore, ectopic expression of TRAF2 in the FASL-sensitive pancreatic cancer cell line Colo357 confers resistance towards FAS-mediated apoptosis, arguing for a pivotal role of TRAF2 in mediating FAS resistance of pancreatic cancer cells. Mechanistically TRAF2 increases as well as basal as FAS-induced NF-κB activity [37]. In addition, TRAF2 overexpression increases the invasive properties of pancreatic cancer cells by inducing the expression of proteolytic enzymes, like matrix metalloproteinases 2 and 9 or the urokinase type plasminogen activator (uPA) [37].

Another strategy to evade death-receptor apoptosis is the overexpression of the decoy receptor 3 (DcR3). Decoy receptors are members of the TNF-receptor superfamily, but without an intact death domain. DcR3 competes with FAS for FASL binding and therefore interferes with FAS triggered apoptosis [35].

Since PDAC cells are type II cells, death receptor induced apoptosis is also blocked at the level of the mitochondria [22]. Furthermore, XIAP contributes to death receptor resistance in PDAC cells [36,38].

### BCL-2 family and PDAC – In favor for survival

At the level of the mitochondria deregulated expression of members of the BCL-2 protein family causes apoptotic resistance in PDAC [2,39,40]. This protein family consists of two pro-apoptotic subgroups, the BAX-like group (BAX, BAK, BOK) and the BH3-only subgroup (BAD, BIK, BID, BIM, BMF, HRK, NOXA, PUMA) and the anti-apoptotic subgroup (BCL-2, BCL-X_L_, BCL-W, MCL-1, A1) [11,14,41]. In most cases, the mitochondrial cell death pathway needs as well as the BAX-like group as the BH3-only group. Here, BH3-only proteins act as sensors of cellular stress, directly antagonizing anti-apoptotic BCL-2 members and activated BAX-like proteins are permeabilizing the outer mitochondrial membrane [14]. In contrast to various other human tumors, expression of the prototypical pro-survival protein BCL-2 is normal or even decreased in PDAC [39,40,42]. Faint BCL-2 expression was detected especially in well-differentiated PDACs. Furthermore, an inverse correlation between BCL-2 and p53 immunoreactivity was observed [40].

Resistance towards apoptosis at the mitochondrial level in PDAC is conferred by at least two anti-apoptotic BCL-2 family members, BCL-X_L _and MCL-1. Both proteins are known to be overexpressed [22,40,43,44]. BCL-X_L _and MCL-1 are upregulated by MAPK signaling in PDAC cells [43]. Whether MCL-1 is also induced by the NF-κB pathway in pancreatic cancer cells, as it has been shown for BCL-X_L_, is unknown at the moment [43,45]. In a murine model of pancreatic cancer, the BCL-X_L _promoter was shown to integrate the epidermal growth factor receptor (EGF-R) signal. Here, EGF-R signaling contributes to the activation of the transcription factors NF-κB and STAT3, both activating the BCL-X_L _gene [45]. In line, inhibition of both transcription factors is needed, to induce significant apoptosis in this model [45].

Functionally, MCL-1 was recently linked to the integrative stress response [46]. Therefore, high MCL-1 might protect pancreatic cancer cells from hypoxia and oxidative stress during tumorigenesis. From a therapeutically viewpoint, dependency of survival of pancreatic cancer cells onto MCL-1 has to be kept in mind, when molecular targeted therapies, like ABT-737 treatment, are considered. ABT-737 is a BAD homologous BH3-mimetic binding with high affinity to BCL-X_L_, BCL-2 or BCL-w but not MCL-1 [47]. Hence, a molecular ABT-737 therapy is ineffective in cells expressing significant amounts of MCL-1 [48]. This applies also for pancreatic cancer cells, where only minor apoptosis was observed after ABT-737 treatment. In contrast, ABT-737 induces distinct apoptosis of pancreatic cancer cells after the depletion of MCL-1 [49]. A further approach to sensitize towards ABT-737 is activation of NOXA, a BH3-only selectively binding to and depleting MCL-1. Enforced NOXA expression in murine embryonic fibroblasts or the myelomonocytic cell line FDC-P1 increased sensitivity for ABT-737 up to 2000-fold [48]. Regulation of NOXA is not understood in pancreatic cancer, but strategies leading to NOXA activation are of value if a molecular ABT-737 therapy is planned.

Little is known about the contribution of the pro-death BCL-2 family members in PDAC. BAX expression is lowered in around 50% of PDACs [8,39]. In line with an essential function in tumor cell killing, the median survival of BAX positive patients after pancreatic resection is 19 months versus 11 months of BAX negative patients [8]. Since BH3-only activation is a potential therapeutic strategy, future studies have to address regulation of BH3-only expression in PDAC in detail.

### Inhibitors of Apoptosis Proteins

The caspases are the central component of apoptosis. The expression levels of caspases are more or less normal in PDAC cells, but proper activation is impaired by potent inhibitors, called IAPs (inhibitor of apoptosis proteins) [29,33,50]. The IAP family, includes different members like cIAP1, cIAP2, XIAP, NAIP, ML-IAP, ILP2, Livin, Apollon and Survivin and one of the molecular mechanisms of these proteins is to bind caspases and inhibit their activity [51]. For PDAC the overexpression of cIAP2, XIAP and Survivin has been demonstrated [26,27,52-54]. A recent functional and systematic investigation of the IAP family in PDAC conducted by Lemoine's group provide evidence that cIAP2 and XIAP play key roles in anti-apoptotic signaling. Downregulation of cIAP2 and XIAP by RNA interference in PDAC cell lines efficiently induced sensitivity towards cisplatin, doxorubicin or paclitaxel [55]. Importantly, no synergism between downregulation of cIAP2 and XIAP was observed [55]. Furthermore, inhibition of XIAP was shown to sensitize PDAC cells for death receptor signaling, cytotoxic therapies and γ-irradiation [36,38,55-59]. Since small molecule inhibitors exist, which target XIAP function, the so-called XIAP antagonists (Xantags), XIAP is a promising target for novel treatment strategies of PDAC [36,59,60].

In addition, Survivin, the smallest member (16,5 kD) of the IAP protein family contributes to anti-apoptotic signaling in PDAC cells. Survivin is selectively expressed in most human neoplasms including pancreatic cancer, but not in normal adult tissue [27,61,62]. Survivin has been implicated in control of cell division and inhibition of apoptosis, by molecular mechanisms recently summarized in an excellent review by Altieri [63]. The control of cell division might explain the induction of apoptosis observed after downregulation of Survivin expression in PDAC cells, which was not overt after downregulation of cIAP2 and XIAP [64-66]. In addition, downregulation of Survivin by RNA interference sensitizes PDAC cells towards TRAIL- and γ-irradiation-induced apoptosis [65,66]. Interestingly, Survivin is expressed in a cell-cycle-dependent manner in PDAC cells. Low expression was observed at the RB-dependent restriction point in the late G1-phase and this particular cell cycle phase was TRAIL-sensitive [66]. Since cell cycle inhibitors are investigated in clinical trials a combination therapy of G1-phase cell cycle inhibition and death receptor activation might be a feasible approach for the treatment of PDAC.

### NF-κB – a key anti-apoptotic transcription factor in PDAC

**Figure 2:**
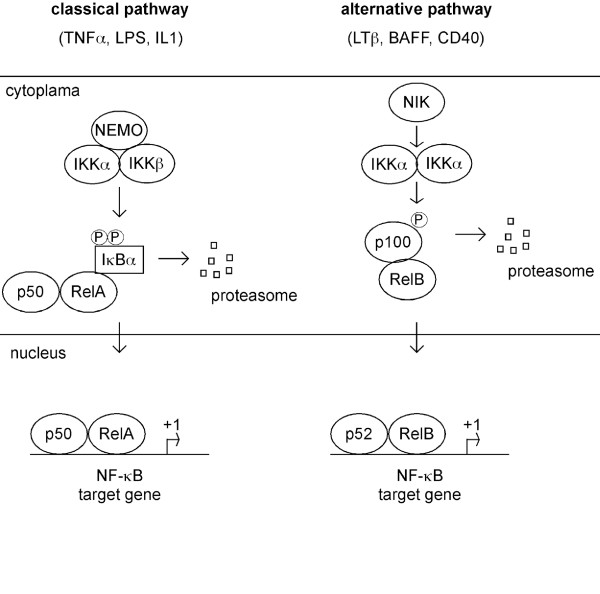
**Representation of the two NF-κB signaling pathways**. Classical signaling (depicted left) is activated by TNFα, IL1β or LPS. Activation of canonical signaling depends on the IKK holocomplex (IKKα, IKKβ, IKKγ/NEMO). Activated IKK phosphorylates IκBs, leading to the proteasomal degradation and subsequent nuclear translocation of classical NF-κB. An alternative signaling pathway (depicted right) is activated by lymphotoxin, CD40L or BAFF. Signaling depends on IKKα and leads to the nuclear translocation of a p52/RelB dimer.

NF-κB is a nuclear transcription factor, which is activated by a variety of different stimuli. NF-κB controls different biological processes like inflammation, cell cycle, apoptosis or epithelial to mesenchymal transition [67]. This transcription factor binds DNA as a dimer, composed of the NF-κB factors RelA (p65), RelB, c-Rel, NF-κB1 (p50/p105) and NF-κB2 (p52/p100). Classical NF-κB (p50/p65 dimer) is usually kept in an inactive form in the cytoplasma by stable association with inhibitor proteins such as IκBα or IκBβ. The IκBs are phosphorylated by a multiprotein kinase complex, the Inhibitor of IκB kinase (IKK). The IKK complex contains the two catalytical kinases IKKα and IKKβ and the structural component IKKγ/NEMO [68,69]. Activation of the IKK complex causes phosphorylation of the IκBs, subsequents ubiquitination and proteasomal degradation [68] (figure 2). This leads to the liberation and nuclear translocation of classical NF-κB and target gene activation. In addition to the canonical NF-κB activation pathway, an alternative pathway involves IKKα-dependent processing of NF-κB2 and nuclear translocation of a p52/RelB dimer (figure 2). The contribution of NF-κB to the tumorigenesis of various cancers is well documented [70,71]. Also in PDAC, NF-κB plays an important role [72-75]. In almost all PDAC cell lines the IKK complex is activated, leading to the nuclear translocation of NF-κB [76-78]. Interference with IKK activity in PDAC cell lines leads to the induction of apoptosis and/or sensitization towards cytotoxic therapies [77,78]. Although IKKα was shown to be overexpressed in PDAC, downregulation of IKKα by RNA interference was not accompanied by significant apoptosis [79,80]. Instead, IKKα was shown to promote cell cycle progression of PDAC cells by inducing transcription of the oncogene S-phase kinase associated protein 2 (SKP2) in a p52/RelB-dependent fashion [80]. Therefore, anti-apoptotic function of NF-κB signaling seems to be mediated by the classical NF-κB pathway, but the contribution of canonical and non-canonical signaling to apoptosis resistance has to be investigated in more detail. In line with activated IKK, the NF-κB subunit RelA/p65 is nuclear translocated in around 45 to 70% of pancreatic cancers [76,77,81]. Furthermore, nuclear RelA/p65 is a predictor for poor patients survival and was linked to a higher expression of NF-κB target genes, like BCL-X_L _[81]. Based on the broad impact of NF-κB signaling towards the apoptotic machinery, it is not surprising that inhibition of NF-κB activity sensitizes pancreatic cancer cells to chemotherapeutic agents and death-receptor mediated apoptosis [82-86]. Target genes of NF-κB in PDAC explaining the sensitizer effect of NF-κB inhibition are BCL-X_L _and c-Flip [82,84-86].

Although NF-κB is activated in PDAC, the signaling pathways leading to IKK activation in PDAC cells are not entirely clear. Here the EGF-receptor-, PI3K-AKT- and K-Ras-pathways were shown to contribute to NF-κB activity [76,77]. Furthermore, NF-κB is modulated by several other important signaling pathways in PDAC. For example, Sarkar's group demonstrated that depleting Notch-1 expression in PDAC cells induces apoptosis and was correlated with reduced NF-κB activity and impaired expression of the NF-κB target gene BCL-X_L _[87,88]. Moreover, Notch-1/NF-κB signaling contributes to invasiveness of PDAC cells [87]. Furthermore, casein kinase 2 (CK2) maintains NF-κB activity and anti-apoptotic signaling in PDAC cells [89]. A further modulator of NF-κB/RelA transcriptional activity is the early response gene IEX-1, which is involved in the regulation of apoptosis. In a recent study, Arlt et al. observed that IEX-1 interacts directly with the C-terminal activation domain of RelA and is involved in inhibition of RelA-dependent transcriptional activity and therefore negatively regulates NF-κB target genes, like Bcl-X_L_, cIAP1 and cIAP2 [90]. This molecular observation might explain the recent demonstration that IEX-1 expression is a significantly favorable factor for survival of patients with PDAC [91].

### The PI3K/AKT survival pathway

The phosphotidylinositol-3' kinase (PI3K)/AKT-pathway is a pro-survival pathway in PDAC cells. This pathway is activated by survival signals like growth factors, cytokines, hormones and oncogenic Ras [92]. Activation of PI3K leads to the phosphorylation of AKT/PKB. Besides the growth promoting potential, activated AKT/PKB favors survival via the direct regulation of apoptotic proteins, like the BCL-2 members BAD and BCL-X_L _or caspase 9 [93-96]. In addition, AKT modulates the activity of transcription factors, like FOXO, NF-κB or c-myc. Schlieman et al. has shown that the PI3K/AKT pathway is activated in about 60% of pancreatic adenocarcinoma tissues and also most pancreatic cancer cell lines display elevated levels of activated AKT [97,98]. Although it was shown that PI3K/AKT signaling modulates NF-κB and c-myc activity in PDAC cells, the molecular activation of this pathway in PDAC is not entirely clear [77,99,100]. Here, the contribution of the tumor suppressor phosphatase and tensin homolog deleted in chromosome ten (PTEN) has been demonstrated [100]. Low expression of this natural antagonist of PI3K signaling was demonstrated in 75% of PDACs [100]. In addition, a receptor tyrosin-kinase is involved in PI3K activation, since the insulin receptor substrate 1 (IRS-1) was shown to be an essential mediator of PI3K activation in quiescent PDAC cells [101]. Furthermore, an unexpected pathway mediates the activation of PI3K-AKT signaling in pancreatic cancer cells. This pathway is regulated by the architectural transcription factor HMGA1. HMGA1 is highly overexpressed in PDAC and known to account for unrestrained proliferation of pancreatic cancer cells [102,103]. Recently, it was shown that HMGA1 activates PI3K-AKT signaling in PDAC cells [104,105]. By forming a complex with the transcription factor C/EBPbeta, HMGA1 activates the insulin receptor gene and the insulin receptor contributes to the activation of PI3K-AKT signaling [106]. This pathway seems to mediate therapeutic resistance, since HMGA1-mediated gemcitabine resistance depends onto intact AKT signaling [107].

Although we observed no overt apoptosis in PDAC cells treated either with the PI3K inhibitor Ly294002 or by interference with AKT1 expression using siRNAs (unpublished data), inhibition of the PI3K/AKT signaling sensitizes PDAC cells towards therapeutic induction of apoptosis [97,108-111]. Since very high concentrations of the PI3K inhibitor Ly294002 (up to 100 μM) were used in many studies and contribution of the PI3K/AKT signaling towards gemcitabine resistance were not detected in BxPc-3, Capan-1 and PancTu-1 cells, the impact of PI3K/AKT signaling for therapeutic resistance of PDAC cells has to be clarified in more detail using functional genomics [112].

## Conclusion

Deregulation of the apoptosis machinery and evasion of apoptosis is a general mechanism in PDAC. Since nearly every step during carcinogenesis activates apoptosis, the development of anti-apoptotic strategies must be an early and essential event. Contribution of molecules like cIAP2 or Survivin to early steps in the carcinogenesis of PDAC should be investigated in relevant mouse models of PDAC in the future. Also therapeutic resistance is mediated by the profound deregulation of the apoptotic machinery in PDAC cells. Detailed molecular knowledge of the anti-apoptotic mechanisms of PDAC cells is essential for the improvement of conventional chemotherapies ("sensitizer strategies") and the development of new potent targeted therapies. Hopefully, this will lead to an improvement of the prognosis of this dismal disease.

## Competing interests

The authors declare that they have no competing interests.

## Authors' contributions

RH: drafted and wrote the manuscript. RMS: drafted and revised the manuscript critically for important intellectual content. DS: drafted and revised the manuscript critically for important intellectual content. GS: drafted and wrote the manuscript, revised the manuscript critically for important intellectual content, given final approval of the version to be published. All authors read and approved the final manuscript.
